# The microgenderome in migraine: integrating sex hormones, microbial composition, and metabolic profiles into a sex-related framework

**DOI:** 10.3389/fmicb.2026.1891150

**Published:** 2026-08-13

**Authors:** Jie Fu, Xianli Liu, Dianzun Liu, Kaiyu Shen, Ming Yao, Huadong Ni

**Affiliations:** Department of Anesthesiology and Pain Research Center, Jiaxing University Affiliated Hospital, The First Hospital of Jiaxing, Jiaxing, Zhejiang, China

**Keywords:** estrobolome, gut-brain axis, microgenderome, migraine, sex differences

## Abstract

**Background:**

Migraine is a debilitating neurological disorder with marked sex differences, as women experience a prevalence two to three times higher than men. Although the gut–brain axis has been implicated in migraine biology, the intersection of sex hormones, microbial signaling, and microbial metabolites remains underexplored. This review evaluates migraine-specific and mechanistically relevant evidence on sex-related microbial and metabolic profiles to examine their potential relevance to sex-related characteristics of migraine.

**Methods:**

This narrative review was informed by a structured literature search of PubMed through May 2026, identifying 22 relevant studies, including clinical studies, interventional studies, animal models, and mechanistic investigations. Given heterogeneity in study design, sequencing methods, sampling strategies, clinical populations, and outcome measures, evidence was synthesized narratively to develop a hypothesis-generating framework for interactions among sex hormones, microbiota, microbial metabolites, and migraine.

**Results:**

Current evidence suggests that gut microbial diversity and composition may be altered in migraine, but sex-stratified migraine-specific data remain limited. Female migraine cohorts have shown reduced abundances of taxa such as *Clostridia* and *Ruminococcus*, alongside enrichment of pro-inflammatory genera including *Desulfovibrio* and *Gemmiger*, with reported associations with inflammatory markers such as interleukin-6 and lipopolysaccharide. Metabolic studies suggest alterations in the tryptophan–kynurenine pathway, including kynurenic acid/quinolinic acid-related changes, whereas direct evidence for sex-specific short-chain fatty acid or neuroactive metabolite profiles in migraine remains limited. Mechanistically, the estrobolome provides a biologically plausible but incompletely validated link through which sex hormones and microbial enzymes may interact with hormone metabolism, gut barrier function, inflammatory tone, and neuroimmune signaling.

**Conclusion:**

The sex hormone–microbiota–metabolite–brain axis should be considered a hypothesis-generating framework rather than an established causal mechanism for migraine sex differences. Future research should use sex-balanced longitudinal cohorts, menstrual-cycle-aware sampling, migraine-specific models, and integrated multi-omics to validate candidate pathways and determine whether microbiome-related signatures can inform sex-stratified biomarkers or adjunctive microbiome-targeted interventions.

## Highlights

- Migraine-associated alterations in gut microbial composition have been reported, but direct sex-stratified evidence remains limited.
- KYNA/QUIN-related changes and short-chain fatty acid pathways represent candidate metabolic signals requiring validation in sex-balanced migraine cohorts.
- The sex hormone–microbiota–metabolite axis is proposed as a hypothesis-generating framework rather than an established causal mechanism of migraine sex differences.

## 1 Introduction

Migraine transcends its characterization as a simple paroxysmal headache, representing a formidable global public health challenge. Currently affecting approximately 14% of the global population, migraine is classified by the World Health Organization as the third leading cause of disability, imposing a substantial socio-economic burden on both individual families and global healthcare systems (Ashina et al., 2021). Clinically, the disorder is characterized by moderate-to-severe unilateral pulsating headaches, frequently accompanied by autonomic symptoms such as nausea, vomiting, and sensory hypersensitivity to light and sound (Ferrari et al., 2022; Shah and Ailani, 2025). While the pathophysiology remains highly complex, current evidence links migraine to activation of the trigeminovascular system (Tohyama et al., 2025), release of neuropeptides such as calcitonin gene-related peptide (CGRP) (Al-Khazali et al., 2023; Christiansen et al., 2025), neuroinflammation (Chen et al., 2022; Lu et al., 2026), and dysregulation of central pain-processing pathways in the brain (Baggio et al., 2024; Mangutov et al., 2025).

Among these complex clinical features, marked sex differences are among the most prominent characteristics of migraine. Epidemiological data indicate that the prevalence in women is two to three times higher than in men (Edvinsson, 2022). A large-scale study involving nearly 30,000 individuals quantified this disparity, reporting a prevalence of 11.43% in women compared to 3.75% in men (Peles et al., 2022) . Beyond the higher prevalence, female patients typically experience a more severe disease course, characterized by increased attack frequency, greater pain intensity, and longer durations of disability (van Casteren et al., 2021; Verhagen et al., 2023). In fact, women bear nearly 80% of the global migraine disease burden (Onan et al., 2023). These sex-related differences have been associated with cyclical fluctuations in ovarian hormones, particularly estrogen, across the female lifespan, including menstruation, pregnancy, and menopause (Warfvinge et al., 2026). However, hormonal fluctuation alone is unlikely to fully explain migraine sex differences, suggesting that additional biological systems may interact with endocrine, immune, metabolic, and nociceptive pathways.

This sex-related clinical pattern is not confined to the nervous system but may also involve the gastrointestinal tract. Nausea and vomiting, as typical accompanying symptoms, are not only included in the diagnostic criteria but may also interfere with the absorption of orally administered medications (Aurora et al., 2021). Furthermore, migraine patients exhibit a high comorbidity with functional gastrointestinal disorders such as irritable bowel syndrome, which has been associated with higher attack frequencies and increased pain severity (Affaitati et al., 2025; Huang et al., 2026). Increased intestinal permeability and chronic mucosal immune activation have been reported in subsets of migraine patients, suggesting that disturbances in the gut microenvironment may contribute to migraine biology beyond the central nervous system, although the precise mechanisms remain incompletely understood (Papetti et al., 2024; Vuralli et al., 2024a; Vuralli et al., 2024b).

These clinical observations have redirected research attention toward the gut–brain axis. Current evidence suggests that migraine may be associated with gut microbial dysbiosis and alterations in microbiota-derived metabolites, including tryptophan-related metabolites and short-chain fatty acids (SCFAs) (Cho et al., 2026; Liu J. et al., 2024; Mugo et al., 2025) . Mendelian randomization studies (He et al., 2023; Ma et al., 2026) and clinical trials involving probiotic supplementation or dietary modification (Fan et al., 2026; Ghavami et al., 2021) have provided preliminary evidence of associations between gut microbial pathways and migraine susceptibility or symptom modulation. However, despite extensive independent investigations into sex differences and the gut-brain axis, research at their intersection remains limited.

Biological sex may influence gut microbial composition, metabolic activity, and host immune regulation, a phenomenon conceptualized as the microgenderome (Bahar et al., 2026; Wu et al., 2024). Distinct differences in microbial pathways and metabolic functions have been reported between males and females even after controlling for age and diet (Vriend et al., 2024), and these sex-related microbial characteristics may influence host immune regulation and metabolic homeostasis (Dodd and Menon, 2022; Gao et al., 2021). Although the established term “microgenderome” is retained from the microbiome literature, the present review focuses primarily on biological sex, sex hormones, reproductive stage, and hormone-related variables rather than sociocultural dimensions of gender. However, direct evidence linking sex-related microbial features to migraine remains limited, as most published migraine microbiome studies have not performed sex-stratified analyses.

In this context, investigating interactions between sex hormones and the gut microbiota may provide a useful framework for understanding migraine sex differences. Sex hormones can reshape microbial architecture, whereas gut bacteria can participate in sex hormone metabolism through specific enzymatic systems, establishing a bidirectional host–microbiota interaction (Leao et al., 2025; Wu et al., 2024). Considering this dimension may help clarify why hormonal fluctuation alone does not fully explain migraine sex differences. Accordingly, this review integrates current evidence regarding sex-related microbial signatures, microbial metabolites, and neuroimmune interactions in migraine while distinguishing direct migraine evidence from mechanistic evidence derived from related disorders and experimental models. Rather than presenting the sex hormone–microbiota–metabolite–brain axis as an established causal mechanism, we evaluate the current level of evidence and propose a hypothesis-generating framework to guide future sex-stratified mechanistic studies, biomarker research, and microbiome-based adjunctive interventions.

## 2 Methods

### 2.1 Evidence acquisition and review strategy

To identify evidence relevant to sex differences in gut microbiota, microbial metabolites, and neuroimmune interactions in migraine, this narrative review was informed by a structured literature search. PubMed was the bibliographic database searched from database inception through May 2026. No additional bibliographic databases were searched. Reference lists of eligible articles were screened manually, and GMrepo was queried as a supplementary microbiome data resource for specific populations, including pediatric migraine (Liu J. et al., 2024).

### 2.2 Study selection and information extraction

To ensure objectivity and accuracy in evidence synthesis, two investigators independently extracted data from the included studies. The extracted information spanned multiple dimensions from basic experiments to clinical investigations, including study design (e.g., cross-sectional studies, randomized controlled trials, animal models), sample size and sex distribution, migraine subtypes, detection methods, and key findings. In particular, we highlighted the statistical descriptions of sex differences, changes in the abundance of key microbial taxa, and levels of metabolites (such as short-chain fatty acids and tryptophan metabolites) reported in each study, thereby laying the data foundation for subsequent mechanistic discussions.

Eligible publications included original clinical studies, interventional studies, experimental animal studies, and mechanistic investigations addressing migraine, gut microbiota, microbial metabolites, sex hormones, or biologically relevant sex differences. Reviews, conference abstracts, editorials, duplicate publications, and studies lacking direct relevance to the scope of this review were excluded. Two investigators independently screened titles, abstracts, and subsequently full texts before reaching consensus regarding study eligibility. Following full-text evaluation, 22 studies were considered sufficiently relevant for the final narrative synthesis. Priority was given to studies providing direct migraine evidence, mechanistic insights, or biologically plausible links between sex hormones, microbiota, and migraine-related pathways.

### 2.3 Narrative synthesis and analytical approach

Given the significant heterogeneity in sequencing technologies, sampling strategies, clinical subtype classifications, participant characteristics, and outcome measures across the included studies, a narrative synthesis approach was employed for evidence integration. This review was designed as a narrative review informed by a structured literature search rather than a formal systematic review or meta-analysis. Accordingly, evidence was synthesized qualitatively based on thematic relevance, biological plausibility, and mechanistic consistency rather than through quantitative evidence synthesis.

No formal risk-of-bias assessment, study quality scoring, or quantitative meta-analysis was performed because the primary objective of this review was to summarize and critically evaluate the current evidence rather than estimate pooled effect sizes. Instead, evidence was interpreted according to study design, migraine specificity, biological plausibility, and the strength of mechanistic support.

Human and animal studies were synthesized within the same conceptual framework because direct sex-stratified evidence in migraine remains limited. Clinical studies provide disease-specific observations, whereas experimental animal studies offer mechanistic insights into sex hormone–microbiota interactions that cannot currently be investigated directly in humans. Throughout this review, direct migraine evidence, evidence from related disorders, animal evidence, and hypothesis-generating interpretations are explicitly distinguished to avoid overstating the current level of evidence.

## 3 Results

### 3.1 Study selection and characteristics of included articles

**FIGURE 1:**
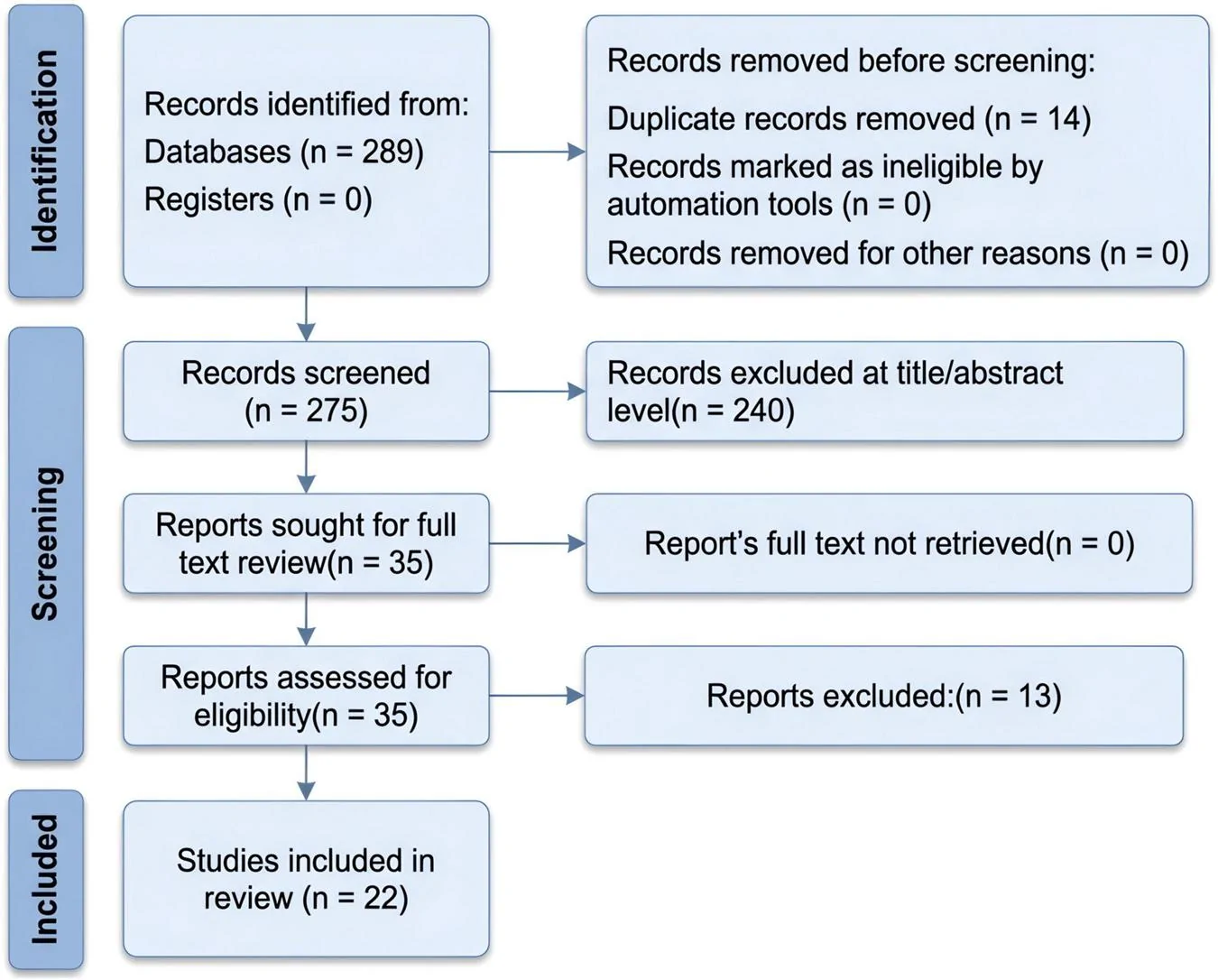
Literature identification and study-selection flow diagram.

**FIGURE 2:**
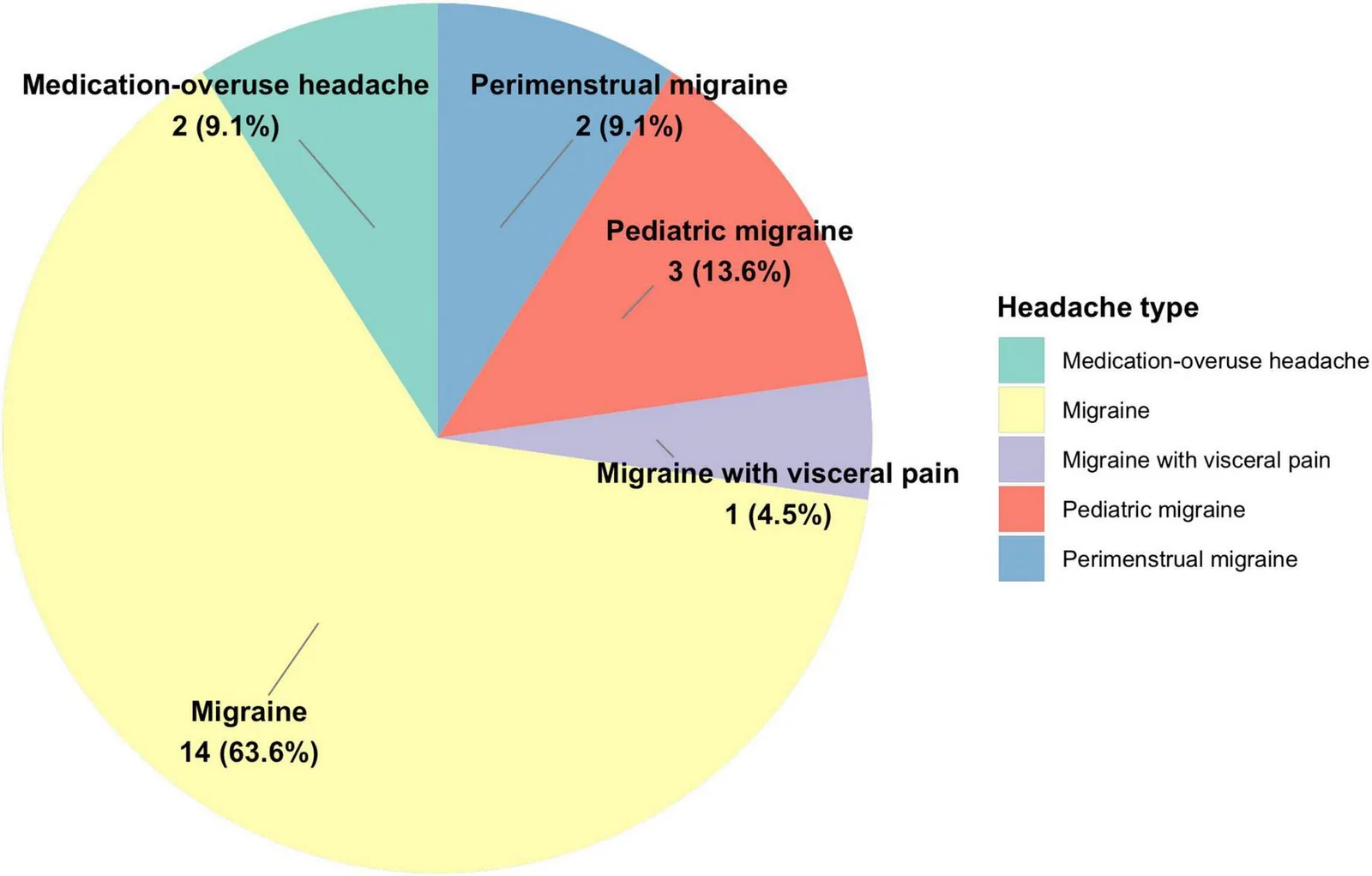
Distribution of Migraine Subtypes.

**FIGURE 3:**
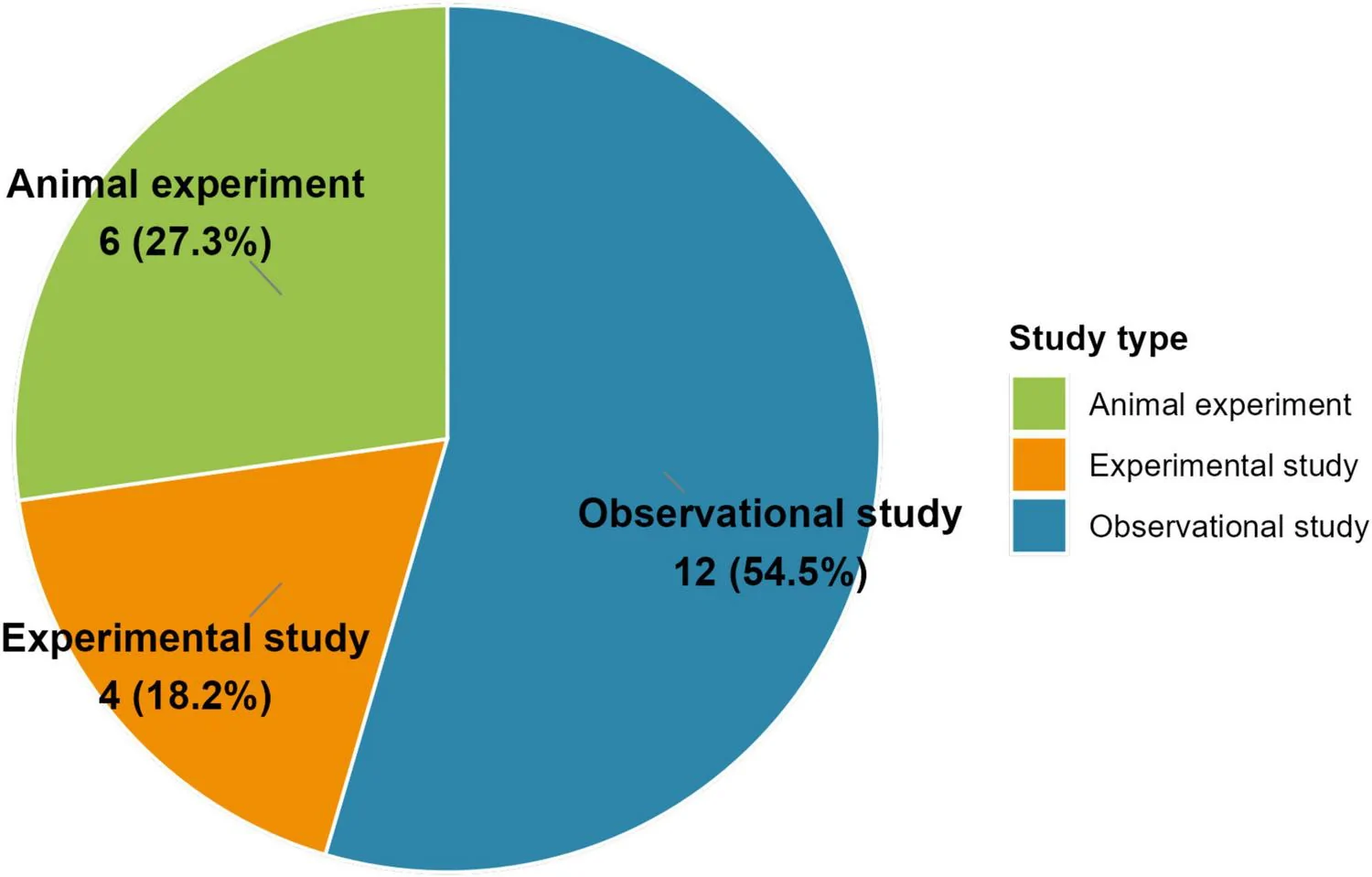
Distribution of Study Types.

Among the 289 articles initially retrieved, 275 highly relevant core records were identified after duplicate removal. Two investigators independently screened the titles and abstracts, followed by a full-text review of potentially eligible studies, resulting in 35 articles after the preliminary screening. These 35 articles subsequently underwent a full-text review, and a total of 22 studies were finally included in the narrative synthesis (Figure 1). The 22 included articles covered five types of headache disorders (Figure 2), comprising 12 observational studies, 4 experimental studies, and 6 animal studies (Figure 3). The main characteristics and findings of studies on sex differences, microbiota, and metabolites in migraine are summarized in Table 1, while the sex distribution and key efficacy outcomes of different interventional strategies for migraine are summarized in Table 2.

**TABLE 1:** Main characteristics and findings of studies on sex differences, microbiota, and metabolites in migraine.

Author/ Year	Species	Sex distribution	Age range	Migraine subtype	Sampling Site	Detection Method	Major microbiota findings	Key metabolite changes	Main findings
Verhagen et al., 2023	Human	1347 Woman, 284 Man	<50 years (Woman: 35.7 ± 9.1 years, Man: 37.4 ± 8.6 years); ≥50 years (Woman: 56.8 ± 5.9 years, Man: 58.0 ± 6.2 years)	Perimenopausal and non-perimenopausal migraine in females; migraine in males	Not applicable	Longitudinal e-diary analysis	Not assessed	Not assessed	Compared with men, women have longer attack durations and more accompanying symptoms for both perimenstrual and non-perimenstrual migraine attacks.
van Casteren et al., 2021	Human	500 Woman	18–80 years	Perimenopausal and non-perimenopausal migraine in females	Not applicable	Longitudinal e-diary analysis	Not assessed	Not assessed	Perimenopausal migraine attacks have longer duration, higher recurrence rate, more severe symptoms, and require more analgesics.
Affaitati et al., 2025	Human	256 Woman	18–58 years	Migraine only vs. migraine with visceral pain	Not applicable	Retrospective observational analysis	Not assessed	Not assessed	Comorbid visceral pain exacerbates migraine; treating visceral pain improves migraine and pain threshold.
Papetti et al., 2024	Human	64 Woman, 34 Man	6–17 years	Pediatric migraine	Feces, urine	16S rRNA, ELISA	Significantly increased bacterial richness and β-diversity indicated dysbiosis in the migraine group	Altered tryptophan and phenylalanine metabolic pathways; elevated urinary indole levels	Gut microbiota and metabolite dysregulation in pediatric migraine patients.
Vuralli et al., 2024a	Human	48 Woman	18–65 years	Chronic migraine(CM)and CM with non-steroidal anti-inflammatory drug overuse headache(CM+NSAID)	Feces, blood	16S rRNA, ELISA	Compared with healthy controls, the CM+NSAID overuse headache group showed lower abundances of Clostridia, *Bacteroidales*, and *Ruminococcus*, but higher abundances of Desulfovibrio, *Gemmiger, Dialister* (genus level) and *Clostridium fessum*, *Blautia luti, Dorea longicatena, Eubacterium coprostanoligenes, Gemmiger formicili*s (species level).	Not assessed	In patients with medication overuse headache, a pro-inflammatory dysbiosis was observed, and alterations in gut microbiota were positively correlated with inflammatory serum markers and headache food triggers.
Liu J. et al., 2024	Human	39 Man, 36 Woman	<10 years	Pediatric migraine	Blood	ELISA	Reduced microbial richness, increased *Bacteroidetes* and *Proteobacteria*, and decreased *Actinobacteria* in children with migraine (data from GMrepo database)	Decreased plasma kynurenic acid, increased serotonin and quinolinic acid; high diagnostic efficacy of kynurenic acid/quinolinic acid ratio	Gut microbiota may play an important role in pediatric migraine pathogenesis by modulating tryptophan metabolism.
Cho et al., 2026	Human	Sex-balanced groups; exact counts not reported in the manuscript summary	19–65 years	Episodic and chronic migraine	Serum	LC–MS/MS	Not assessed	Serum butyrate was lower in untreated episodic and chronic migraine; preventive treatment was associated with higher propionate, particularly in chronic migraine.	SCFA patterns varied with migraine and preventive-treatment status. The cross-sectional findings do not establish directionality or sex-specific metabolic effects.
He et al., 2023; Ma et al., 2026	Human genetic data	Sex-stratified effects not reported	Not applicable	Migraine, migraine with aura, migraine without aura	GWAS summary data	Two-sample Mendelian randomization	Genetically predicted associations were reported for taxa including *Coprococcus, Anaerotruncus, Bifidobacteriales*, and *Actinobacteria.*	Not assessed	The findings provide genetic support for potential microbiota–migraine links but are model-dependent and do not prove clinical causality.
Fan et al., 2026	Human	Sex-stratified effects not reported	6–19 years	Pediatric migraine	Feces, blood	16S rRNA, ELISA	Significant differences in gut microbiota of migraine patients, with decreased *Bifidobacterium* and increased *Bacteroides*	Not assessed	Decreased *Bifidobacterium* longum and increased *Bacteroides* in migraine patients; *B. longum* supplementation is a promising adjunctive therapy for pediatric migraine management.
Gecse et al., 2022	Human	43 Woman	18–50 years	Intermittent migraine without aura	Blood	LC–MS/MS	Not assessed	Elevated baseline tryptophan/large neutral amino acid ratio; no increase in tryptophan or kynurenine after citalopram challenge; negative correlation between kynurenine/tryptophan ratio and attack frequency	Impaired activation of the tryptophan-kynurenine pathway and increased vascular reactivity in migraine patients, offering new drug development targets.
Tuka et al., 2021	Human	84 Woman	25–50 years	Migraine	Blood	UHPLC–MS/MS	Not assessed	Decreased tryptophan, kynurenine, kynurenic acid, etc. (interictal); increased anthranilic acid and melatonin, etc. (ictal)	Widespread suppression of peripheral tryptophan catabolism in migraine patients, potentially inducing glutamate excitotoxicity and triggering attacks.
Wang et al., 2022	Human	43 Woman, 22 Man	14–60 years	Intermittent migraine, chronic migraine, medication-overuse headache	Brain regions assessed by MRS	MEGA-PRESS sequence and 3-Tesla magnetic resonance scanner	Not assessed	Decreased GABA in dentate nucleus and increased Glx in periaqueductal gray matter in chronic migraine patients	Neurotransmitter abnormalities in the dentate nucleus and periaqueductal gray matter pathologically underpin migraine chronification and correlate with clinical symptoms.
Gao et al., 2024	Mouse	Male	8 weeks	Chronic migraine	Brain tissue	^1^H-MRS analysis	Not assessed	Reduced energy metabolism, elevated glutamate, reduced GABA in brain regions of chronic migraine-like mice, most significant in trigeminal nucleus caudalis and thalamus	Energy metabolism dysfunction and excitatory/inhibitory neurotransmitter imbalance are key mechanisms in migraine chronification; the trigeminal nucleus caudalis-thalamus-somatosensory pathway is involved in pain transmission.
Miao et al., 2022	Rat	Male	Not reported	Migraine	Feces, blood	16S rRNA and GC–MS/MS	Dural inflammation-induced migraine model altered gut microbiota, with the most significant marker being a decrease in *Lactobacillus*.	Enhanced microbial metabolic pathways (butyrate, propionate, tryptophan); elevated fecal indole-3-acetamide and tryptophol.	Cephalic allodynia induced by dural inflammation can alter gut microbiota composition and metabolic pathways, suggesting a bidirectional gut-brain axis mechanism.
Cho et al., 2025	Human	151 Woman, 14 Man	19–65 years	Episodic migraine, chronic migraine	Saliva	16S rRNA	Oral dysbiosis in migraine patients (13 genera like *Gemella* altered); weaker gut microbiota changes	Enriched taxa linked to carbohydrate metabolism; depleted taxa linked to nitrogen metabolism (nitrate/nitrite reduction)	Oral dysbiosis may contribute to migraine; specific bacterial genera could serve as diagnostic biomarkers and therapeutic targets.

5-HT, 5-hydroxytryptamine; 16S rRNA, 16S ribosomal RNA; BDNF, brain-derived neurotrophic factor; CM, chronic migraine; ELISA, enzyme-linked immunosorbent assay; GABA, γ-aminobutyric acid; GC–MS/MS, gas chromatography–tandem mass spectrometry; Glx, glutamate plus glutamine; LC–MS/MS, liquid chromatography–tandem mass spectrometry; MEGA-PRESS, Mescher–Garwood point-resolved spectroscopy; NSAIDs, non-steroidal anti-inflammatory drug;

^1^H-MRS, proton magnetic resonance spectroscopy; TrkB, tropomyosin receptor kinase B; UHPLC–MS/MS, ultra-high-performance liquid chromatography–tandem mass spectrometry.

**TABLE 2:** Summary of sex distribution and principal results from interventional studies for migraine.

Author/Year	Species	Sex distribution	Main findings
Fan et al., 2026	Human and rat	Human pilot: not reported by sex; rats: male	*Bifidobacterium longum* supplementation was evaluated in an exploratory 23-patient pediatric pilot and in young and adult rat models. Headache outcomes improved in the pilot, and trigeminal activation was attenuated in rats; controlled confirmation and sex-stratified evaluation are required.
Ghavami et al., 2021	Human	80 Woman	Multi-strain biological supplement serves as an adjunctive therapy for female migraine, improving migraine symptoms, inflammatory markers, and gut permeability, thereby reducing disease burden.
Lanza et al., 2021	Mouse	Female	Sodium propionate and butyrate alleviate migraine-like hyperalgesia by modulating gut microbiota, suggesting short-chain fatty acids as potential therapies for migraine-related gut dysfunction.
Liu J. et al., 2024	Rat and mouse	Male	Tianshu capsule’s small molecules exert anti-migraine effects via antioxidation; its polysaccharides act by modulating microbiota, short-chain fatty acids, and tryptophan metabolism without being absorbed.
Wang et al., 2026	Rat	Female	Puerariae Lobatae Radix - Gastrodiae Rhizoma reverses 52 plasma metabolites (e.g., tauroursodeoxycholic acid) and restores glutamate, GABA, and lipid levels in the prefrontal cortex-thalamus circuit.
Vajdi et al., 2024	Human	80 Woman	Inulin supplementation positively affects migraine symptoms and mental health in females with migraine.
Tereshko et al., 2023	Human	60 Woman	Both 2:1 ketogenic and low-glycemic-index diets improve migraine symptoms and disability.

GABA, γ-aminobutyric acid; SCFAs, short-chain fatty acids.

### 3.2 Gut microbiota characteristics under migraine sex differences

#### 3.2.1 Sex-dependent variations in microbial diversity

Microbial diversity is commonly used to characterize the global structure of the gut microbiota. Alpha diversity (α-diversity) reflects microbial richness and evenness within individuals, whereas beta diversity (β-diversity) captures differences in microbial community composition between individuals. These indices provide an overview of microbial ecosystem structure and are frequently used as initial indicators of gut microbial dysbiosis. In migraine research, these measures may be biologically relevant because changes in microbial richness or community structure may reflect altered microbial functional capacity for metabolite production, intestinal barrier regulation, and immune signaling; however, diversity metrics alone do not identify a specific disease mechanism.

In migraine, preliminary studies suggest that gut microbial diversity may be altered. A 16S rRNA sequencing study of children aged 6–17 years reported increased bacterial richness in patients with migraine, together with significant differences in β-diversity, indicating a distinct microbial community structure (Papetti et al., 2024). Another fecal microbiota analysis in pediatric migraine similarly identified compositional differences based on Shannon and Simpson indices (Liu J. et al., 2024). These findings support a potential association between migraine and gut microbial dysbiosis. However, neither pediatric migraine study performed sex-stratified analyses. These migraine-specific findings therefore support an association between migraine and gut microbial diversity, but they do not establish sex-specific microbial alterations in migraine.

The need for sex-stratified analysis is supported by evidence from non-migraine populations and other disease contexts. In healthy adults, women have been reported to show higher species richness, whereas men exhibit greater β-diversity, which correlates positively with age and physical fitness (Tzemah-Shahar et al., 2024). In middle-aged individuals, α-diversity correlates with skeletal muscle mass index in men but not in women (Park et al., 2022). Sex-related microbial differences may also emerge early in life: compared with female infants, male infants show lower α-diversity, altered community structure, and increased abundance of Enterobacterales (Kennedy et al., 2025). By 12 years of age, modest sex-related differences in β-diversity remain detectable (Ou et al., 2023). In adults aged 50 years and older, mobility disability is associated with reduced α-diversity and altered β-diversity in women, but not in men (Peters et al., 2026).

Disease-related studies further suggest that biological sex may influence the relationship between microbial diversity and host pathophysiology. In essential hypertension, β-diversity was associated with pathophysiological markers related to blood pressure regulation in female patients, whereas no comparable associations were observed in males (Virwani et al., 2023). Among patients with trauma or sepsis, microbial diversity in females remained relatively stable compared with healthy controls, whereas males showed more pronounced dysbiosis and sex-specific fecal metabolite alterations (Munley et al., 2024). In inflammatory gut-related autoimmune conditions, microbial signatures also display sex-differentiated features (Alizadeh et al., 2025). These observations suggest that the clinical meaning of microbial diversity may differ between males and females, although this possibility remains untested in migraine-specific cohorts.

Taken together, migraine studies suggest altered microbial diversity, whereas evidence from non-migraine populations indicates that biological sex can shape diversity metrics and their host associations. Thus, diversity findings in migraine should be interpreted as preliminary evidence of gut microbial involvement rather than proof of sex-specific microbial alterations; their clinical relevance requires validation in well-characterized, sex-stratified migraine cohorts.

#### 3.2.2 Differences in core microbiota composition at the phylum and genus levels

At the phylum and genus levels, biological sex has been associated with differences in the composition of the core gut microbiota. A large-scale cohort study involving 5,166 subjects demonstrated that sex remains significantly associated with gut microbiota composition even after adjusting for factors such as diet, age, and cardiovascular risk (Vriend et al., 2024). These differences are particularly prominent in metabolic pathways related to vitamin B6 synthesis and sucrose degradation, suggesting that biological sex may contribute to baseline variation in gut microbial functional profiles. Although these observations were derived from healthy populations rather than migraine cohorts, they provide an important biological framework for interpreting sex-related microbial variation in migraine.

In the context of migraine research, limited clinical and genetic evidence has suggested alterations in specific bacterial genera. Clinical evidence from female cohorts with chronic migraine has reported reduced abundances of the class *Clostridia* and the genus *Ruminococcus*, alongside enrichment of genera with pro-inflammatory potential, such as *Desulfovibrio*; these microbial alterations have been associated with circulating inflammatory markers, including interleukin-6 (IL-6) and lipopolysaccharide (LPS) (Vuralli et al., 2024a). Although metagenome-wide association studies and Mendelian randomization analyses have identified multiple genera associated with migraine risk, such as *Coprococcus* and *Actinobacteria*, most existing studies have not performed sex-stratified analyses (He et al., 2023; Ma et al., 2026). Therefore, whether these candidate migraine-associated genera contribute differently to migraine risk in male and female patients remains unclear.

Experimental animal studies from non-migraine models provide indirect evidence that sex hormones can influence gut microbial composition and function. For example, androgen-related changes in male rats have been associated with remodeling of the gut microbiota (Li et al., 2025). Evidence from other experimental settings also suggests that sex-related microbial differences may affect metabolic responses, including tryptophan metabolism (Bardhan et al., 2024). These findings support the broader biological premise that hormonal status can modify the gut microbial environment. When considered together with the migraine-associated microbial alterations described above, they provide a rationale for investigating whether sex hormones modify microbial signatures in migraine. However, these non-migraine findings should not be interpreted as direct evidence of a sex hormone–microbiota mechanism in migraine, which remains to be validated in sex-balanced clinical cohorts and migraine-relevant animal models.

In summary, current evidence suggests the presence of sex-related differences in core gut microbiota composition, although direct migraine-specific evidence remains limited and much of the available evidence is derived from non-sex-stratified studies or supporting experimental models. Consequently, future research should prioritize deep sequencing analysis with sex stratification. Precisely identifying the distribution patterns of key taxonomic units, such as the *Firmicutes* to *Bacteroidetes* ratio, *Ruminococcus*, and *Desulfovibrio*, in male and female patients will help clarify whether these microbial characteristics contribute to migraine sex differences and provide a stronger microecological basis for future mechanistic and translational studies.

### 3.3 Sex-specific analysis of metabolite profiles in migraine

#### 3.3.1 Sex-dependent distribution of short-chain fatty acids

SCFAs, including acetate, propionate, and butyrate, are major metabolic products of gut microbial fermentation and have been implicated in gut–brain axis signaling (Dalile et al., 2019; Forte et al., 2024; Hays et al., 2024). Through their effects on immune regulation (Chang et al., 2024), intestinal barrier integrity (Seethaler et al., 2022), neurotransmitter synthesis (Su and Xia, 2026), and blood–brain barrier function (Fock and Parnova, 2023), these metabolites may influence neuroinflammatory and nociceptive processes relevant to migraine (Lanza et al., 2021). Although direct evidence regarding sex-specific SCFA profiles in migraine patients remains limited, current animal and clinical studies provide preliminary support for the involvement of SCFAs in migraine-related biological processes.

A recent clinical study enrolling 476 participants further reported that serum butyrate levels were lower in both episodic and chronic migraine patients not receiving preventive treatment than in healthy controls, whereas acetate and propionate levels did not differ significantly (Cho et al., 2026). In patients with chronic migraine, preventive treatment was independently associated with higher serum propionate levels, while among participants receiving preventive treatment, higher butyrate levels were linked to more headache days per 30 days (Cho et al., 2026). In a rat model of migraine, the polysaccharide component of Tianshu capsules modulated colonic levels of branched-chain and straight-chain fatty acids, accompanied by improved tryptophan-related metabolic profiles and attenuated migraine-like behaviors (Liu J. et al., 2024). In a nitroglycerin-induced migraine mouse model, sodium propionate and sodium butyrate treatment attenuated migraine-like pain behaviors and photophobia, reduced histological damage in the trigeminal nucleus, and decreased the expression of pro-inflammatory mediators (Lanza et al., 2021).

While the aforementioned evidence is primarily derived from non-sex-stratified animal models, mixed-sex cohorts, or studies without detailed sex-stratified reporting, available evidence suggests that SCFA production and utilization may be influenced by biological sex. Experimental studies suggest that estrogen may influence gut microbial composition and SCFA-producing pathways (Tang et al., 2025). Given the higher prevalence and disease burden of migraine in women, sex-related differences in SCFA metabolism may represent a biologically plausible hypothesis linking microbial metabolism to differences in pain processing. However, direct clinical evidence supporting this hypothesis in migraine patients remains limited. Consequently, future investigations should use sex-stratified metabolomics to characterize major SCFAs, including acetate, propionate, and butyrate, across male and female patients and examine their associations with clinical phenotypes, hormonal status, and treatment exposure in migraine.

#### 3.3.2 Sex-specific characteristics of the tryptophan metabolism pathway

**FIGURE 4:**
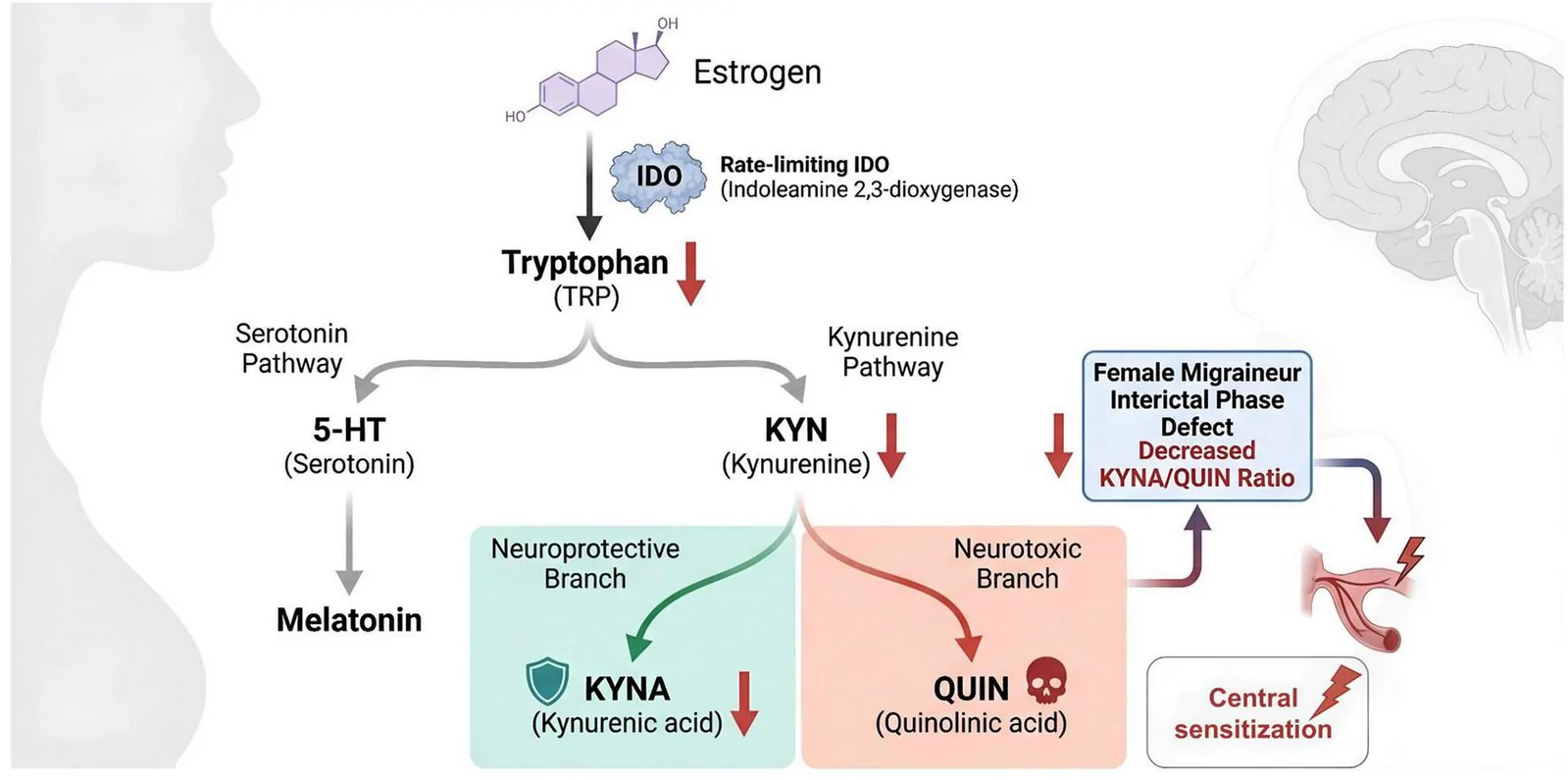
Reported alterations in tryptophan–kynurenine metabolism in female and pediatric migraine cohorts. Tryptophan is metabolized through serotonergic and kynurenine pathways. Reduced kynurenic acid (KYNA) levels and altered KYNA/quinolinic acid (QUIN)-related measures have been reported in selected female and pediatric migraine cohorts. Hormonal modulation of indoleamine 2,3-dioxygenase (IDO) activity represents a biologically plausible hypothesis, but a sex-specific metabolic shunt and its direct contribution to central sensitization have not been established in migraine. Illustration was created with Biorender.com.

Tryptophan (TRP) metabolism and its downstream pathways have been increasingly implicated in migraine pathophysiology. This essential amino acid undergoes biotransformation through two primary routes: the synthesis of the neurotransmitter serotonin (5-HT) and the kynurenine (KYN) pathway, which generates various neuroactive metabolites (De la Fuente Muñoz et al., 2023). Current clinical evidence suggests that this pathway is altered in migraine, particularly in female and pediatric cohorts (Figure 4).

In female patients with migraine without aura, an elevated baseline ratio of TRP to large neutral amino acids has been reported. After acute citalopram challenge, these patients failed to show the expected increases in TRP and KYN observed in healthy controls, suggesting altered regulation of the TRP–KYN pathway in this specific patient population (Gecse et al., 2022). Further clinical metabolic profiling has demonstrated dynamic alterations in tryptophan-related metabolites across different phases of migraine. Specifically, metabolic profiling in female migraineurs aged 25–50 years showed reduced plasma levels of TRP, KYN, KYNA, indole-3-acetic acid, and melatonin during the interictal phase, with partial normalization during attacks (Tuka et al., 2021). Because these observations were obtained exclusively from women, their applicability to male migraine patients remains unknown. Notably, KYNA levels correlate significantly with headache severity and attack frequency. Similar metabolic signatures are observed in pediatric populations: reduced plasma KYNA together with increased 5-HT and QUIN has been reported in pediatric migraine, and the KYNA/QUIN ratio showed preliminary diagnostic performance (Cho et al., 2026). However, these pediatric findings should be interpreted independently from adult female studies because age, developmental stage, and maturation of tryptophan metabolism may substantially influence metabolite profiles. Collectively, these studies suggest that dysregulation of the tryptophan–kynurenine pathway may be involved in migraine. However, current evidence remains insufficient to determine whether these metabolic alterations represent sex-specific biological characteristics or more general features of migraine.

The sex-related differences observed in tryptophan metabolism is likely linked to the hormonal regulation of key rate-limiting enzymes. Experimental evidence suggests that estrogen levels significantly influence the activity of indoleamine 2,3-dioxygenase (IDO), thereby altering the KYN/TRP ratio and the subsequent distribution of metabolites (Luo et al., 2025; Sriprasert et al., 2025). This hormone-enzyme-microbiota interaction represents a biologically plausible mechanism that may contribute to sex differences in migraine susceptibility and progression; however, its relevance in human migraine remains to be directly validated. Moreover, most current studies on tryptophan metabolism remain limited to single-sex cohorts or non-stratified mixed populations, restricting the ability to determine whether observed metabolic alterations are truly sex-specific.

#### 3.3.3 Neuroactive substances: potential sex-related mechanisms

An imbalance between excitatory and inhibitory neurotransmission, particularly involving γ-aminobutyric acid (GABA) and glutamate, has been implicated in central sensitization and migraine chronification (Li et al., 2024; Peek et al., 2021). Although GABA and glutamate are primarily studied within the central nervous system, gut microorganisms may influence neuroactive signaling indirectly through microbial metabolites, immune modulation, vagal afferent pathways, and effects on gut and brain barrier integrity (Belelli et al., 2025; Chen et al., 2025; Pan et al., 2025). Importantly, peripheral GABA does not readily cross the blood–brain barrier, indicating that microbiota-related GABAergic effects are more likely to occur through indirect gut–brain communication pathways rather than direct transfer of peripheral GABA into the brain.

Existing neuroimaging evidence indicates that GABA levels are significantly reduced in specific brain regions, such as the dentate nucleus, in patients with chronic migraine (Chen et al., 2024). Conversely, glutamate levels are elevated in the periaqueductal gray, and these alterations correlate closely with sleep quality and pain perception (Peek et al., 2021). Animal models further support the involvement of excitatory–inhibitory imbalance in migraine-like chronification (Gao et al., 2024). Although most of these studies have not performed sex-stratified analyses, the involvement of the gut microbiota offers a novel perspective for explaining potential sex differences. Specific gut microbes, including members of the genera *Lactobacillus* and *Bifidobacterium*, possess the enzymatic capacity to synthesize GABA, and the abundance of these taxa varies significantly between sexes (Tamés et al., 2023; Zhong et al., 2023; Zou et al., 2024). In female rat models, interventions targeting neurotransmitter pathways demonstrate higher sensitivity, suggesting that female individuals may possess unique biological feedback mechanisms for regulating gut-derived neuroactive substances (Wang et al., 2026).

Potential sex-related differences in GABAergic and glutamatergic signaling may reflect interactions among sex hormones, the gut microbiota, and host neuroimmune pathways. Estrogen and progesterone have been reported to influence receptor expression and signaling within the central GABAergic system (Ingram et al., 2022; Wu et al., 2025), while hormone-related alterations in gut microbial composition may modify microbial neuroactive signaling through indirect gut–brain communication pathways. These interactions may vary across different phases of the menstrual cycle; however, direct evidence linking microbiota-related GABA or glutamate metabolism to sex differences in migraine remains limited. Quantitative comparisons of neuroactive substances in cerebrospinal fluid, plasma, and intestinal samples between male and female patients are also scarce. Future studies integrating imaging metabolomics with metagenomic data should test whether these pathways are associated with central sensitization in sex-stratified migraine cohorts.

### 3.4 Sex-specific interaction mechanisms among microbiota, metabolites, and the nervous system in migraine

#### 3.4.1 Bidirectional regulation between sex hormones and the gut microbiota

**FIGURE 5:**
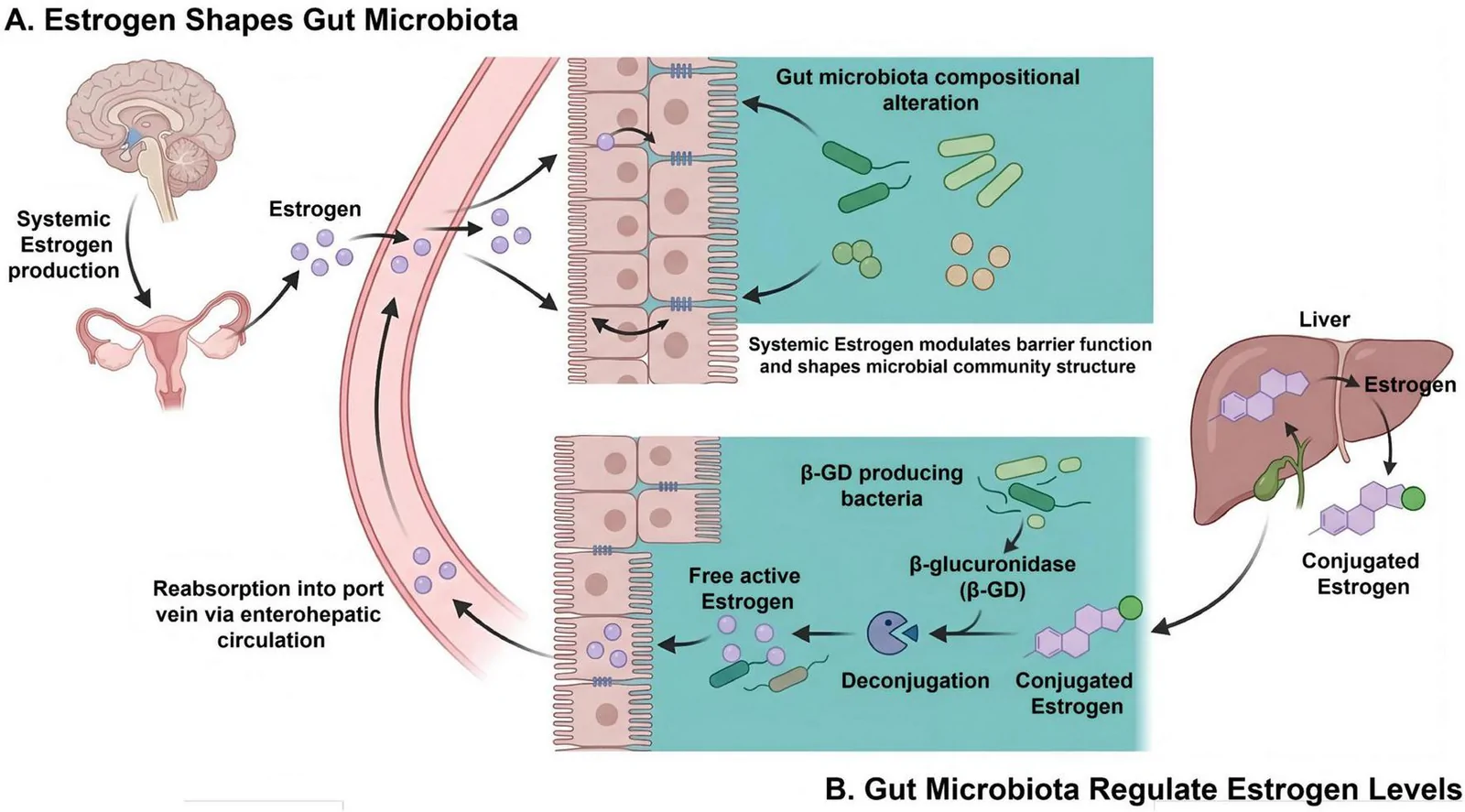
Bidirectional regulation between sex hormones and gut microbiota in migraine. **A** Systemic estrogen modulates intestinal barrier function, and shapes gut microbial community structure; **B** Gut bacteria, especially β-glucuronidase (β-GD)-producing taxa, deconjugate conjugated estrogen in the intestine, liberating free active estrogen that is reabsorbed into the portal vein via enterohepatic circulation, thereby regulating systemic estrogen levels. Illustration was created with Biorender.com.

A bidirectional relationship between sex hormones and the gut microbiota has been increasingly recognized (Hokanson et al., 2024). Host sex hormones, particularly estrogen, may influence gastrointestinal motility, epithelial barrier integrity, mucosal immune activity, and the structural composition of gut microbial communities (Guo et al., 2023; Hokanson et al., 2024; Moadi et al., 2024). Conversely, gut microorganisms can participate in the deconjugation and enterohepatic circulation of sex hormones through enzymatic systems such as β-glucuronidase (β-GD) (Leao et al., 2025; Wu et al., 2024). This process may modulate the concentration of bioactive hormones in the systemic circulation and is commonly regarded as a functional component of the estrobolome (Figure 5).

This pathway may be relevant to migraine because attacks in women often cluster around periods of rapid hormonal transition, including menstruation, perimenopause, and menopause (Peng et al., 2025; Raffaelli et al., 2023; van Casteren et al., 2021). Altered estrobolome activity may therefore represent a biologically plausible bridge between hormonal fluctuations and gut–brain axis dysfunction. However, the estrobolome has not been directly validated as a migraine-specific mechanism, and current support is based primarily on the established association between hormonal fluctuations and migraine and on the known capacity of gut microbiota to regulate estrogen metabolism. Accordingly, the estrobolome should be regarded as a hypothesis-generating functional link rather than an established mechanism underlying migraine sex differences.

#### 3.4.2 Gut barrier dysfunction and sex-related neuroimmune activation

Gut dysbiosis may contribute to migraine sensitization through intestinal barrier dysfunction, low-grade systemic inflammation, and neuroimmune activation, but the extent to which these pathways differ by biological sex remains incompletely defined (Papetti et al., 2024; Wijeratne et al., 2025; Zhang et al., 2024). In female patients with chronic migraine and medication-overuse headache, reduced *Clostridia* and enriched *Desulfovibrio* have been associated with elevated inflammatory markers, including IL-6, high mobility group box 1 (HMGB1), and LPS (Vuralli et al., 2024a). These findings support the plausibility of a microbiota–endotoxin–inflammation pathway in female migraine cohorts, although they do not establish sex-specific causality. Interventional studies provide additional, although still preliminary, support for the relevance of gut barrier and inflammatory pathways. In a randomized double-blind trial, a 12-week synbiotic intervention in women with migraine reduced attack frequency and serum high-sensitivity C-reactive protein (hs-CRP) levels, with improvement accompanied by changes in the intestinal permeability marker zonulin (Ghavami et al., 2021). These findings suggest that zonulin may serve as an exploratory marker linking gut barrier function, inflammatory tone, and migraine outcomes, although its specificity for sex-stratified migraine mechanisms remains to be established.

Mendelian randomization studies provide genetic evidence for potential links among gut microbial taxa, inflammatory mediators, and migraine risk; however, most analyses have not established sex-biased effects. Several gut taxa, including *Actinobacteria*, have been associated with migraine susceptibility, with circulating inflammatory proteins such as IL-6 and tumor necrosis factor-alpha (TNF-α) proposed as possible mediators; however, these mediation estimates should be considered preliminary and model-dependent (Ma et al., 2026). Experimental animal models provide complementary mechanistic clues but should not be regarded as direct validation of migraine-specific or sex-biased causal pathways. For example, in a maternal immune activation model, transplantation of microbiota from male donors exacerbated neuroinflammatory manifestations in male recipient mice, whereas microbiota from female donors did not produce comparable effects (Salia et al., 2025). This finding suggests that gut microbial signals may have sex-dependent neuroimmune consequences in non-migraine experimental settings. These observations illustrate the broader principle that host biological sex can modify microbiota-related immune responses, but their relevance to migraine requires disease-specific validation.

Within a microgenderome framework, the key question is not only whether gut barrier dysfunction and inflammation occur in migraine, but whether these pathways are modified by biological sex, hormonal status, medication exposure, or estrobolome activity. This possibility remains biologically plausible but not yet established and should be evaluated alongside intestinal permeability, endotoxemia, systemic inflammation, hormone profiles, medication use, and clinical phenotypes in future sex-stratified migraine studies.

#### 3.4.3 Potential sex-related regulation of gut microbiota-derived metabolites

Gut microbiota-derived metabolites may influence migraine-related pain processing through pathways involving tryptophan metabolism, SCFAs, serotonergic signaling, and neuroactive amino acid metabolism (De la Fuente Muñoz et al., 2023; Lanza et al., 2022; Zhang et al., 2025). Alterations in tryptophan–kynurenine metabolism may involve changes in the balance between neuroprotective and potentially excitatory or neuroactive downstream metabolites. Clinical studies have reported reduced KYNA and increased 5-HT and QUIN in specific migraine cohorts, including pediatric migraine and female migraine populations (Liu J. et al., 2024; Tuka et al., 2021). Accordingly, the KYNA/QUIN ratio may represent an exploratory indicator of the balance between neuroprotective and excitatory kynurenine metabolites, with potential relevance to migraine-related neurochemical profiling (Liu J. et al., 2024). Serotonergic signaling is an important component of gut–brain communication and pain modulation, and experimental evidence suggests that sex hormones and microbial signals may modify serotonergic tone and behavioral responses (Xu et al., 2025).

Mechanistic studies provide indirect support for a microbiota–vagus nerve–brainstem pathway that may participate in pain modulation. Animal studies suggest that microbial alterations may signal through the vagus nerve to influence brainstem nuclei, including the nucleus tractus solitarius and dorsal raphe nucleus, thereby modulating serotonergic activity (Liu et al., 2025). In migraine rat models, reduced *Lactobacillus* abundance has been associated with changes in butyrate, propionate, and tryptophan-related metabolites, including indole-3-acetamide and tryptophol, which may influence trigeminovascular excitability (Miao et al., 2022). However, evidence that these neurochemical effects are sex-specific in migraine remains limited. For instance, non-migraine experimental evidence suggests that microbial metabolites such as indoles may exert sex-dependent effects on physiological indicators, and that tryptophan metabolic responses to external stressors may differ between males and females (Bardhan et al., 2024). These findings provide mechanistic clues for considering biological sex in studies of microbiota-related neuroactive metabolites, but they do not establish a neurochemical basis for migraine sex differences. Gut microbiota-related neuroactive metabolites should not be viewed as sex-neutral by default; however, their sex-modified effects on trigeminovascular signaling remain to be directly tested in migraine-specific studies.

### 3.5 Clinical translation and challenges

#### 3.5.1 Exploratory migraine biomarkers integrating sex dimensions

Sex-related differences in the gut microbiota and microbial metabolites may provide a theoretical basis for exploring candidate migraine biomarkers. Clinical data suggest that tryptophan–kynurenine metabolites, particularly KYNA and QUIN, may have exploratory value for migraine-related metabolic profiling. For instance, in pediatric migraine patients, the plasma kynurenic acid/quinolinic acid (KYNA/QUIN) ratio showed preliminary diagnostic performance, with a reported area under the curve (AUC) of 0.871 and sensitivity and specificity exceeding 80% (Liu J. et al., 2024). Evidence from adolescent populations with major depressive disorder (MDD) also suggests sex-related alterations in kynurenine metabolism, including reduced kynurenic acid concentrations and altered KYNA/QUIN-related indices in females; however, this should be interpreted as indirect evidence from a non-migraine condition rather than direct support for sex-specific migraine biomarkers (Nikkheslat et al., 2025).

Clinical observations in migraine have also reported lower interictal KYNA levels in patients with non-menstrual cycle-related severe headaches, suggesting a possible relationship among hormonal context, tryptophan–kynurenine metabolism, and symptom severity (Tuka et al., 2021). Together, these findings suggest that the tryptophan–kynurenine pathway, especially KYNA/QUIN-related measures, may serve as a candidate metabolic domain for future sex-stratified validation rather than a validated marker of central sensitization.

Beyond systemic metabolites, intestinal barrier markers and oral microbial profiles may provide exploratory information for migraine stratification and candidate biomarker development. Elevated urinary indoxyl has been reported together with increased plasma zonulin and immunoglobulin A (IgA), suggesting increased intestinal permeability and mucosal immune activation (Papetti et al., 2024). Furthermore, machine-learning analysis of oral microbiota profiles has shown exploratory classification performance for identifying migraine patients, with reported AUC values ranging from 0.83 to 0.88 (Cho et al., 2025). These findings suggest that oral microbiome classifiers, when integrated with gut microbial, metabolic, inflammatory, and barrier-related markers, may help construct multidimensional candidate models for future validation.

In developing such candidate models, biological sex should be considered as a potentially important stratification variable. Large-scale cohort studies indicate that biological sex remains associated with gut microbiota composition even after adjustment for age, diet, and cardiovascular risk factors (Vriend et al., 2024). Future models should evaluate whether incorporating biological sex, hormonal status, reproductive stage, microbial signatures, KYNA/QUIN-related measures, zonulin, oral microbiome classifiers, inflammatory cytokines, and clinical phenotypes improve prediction beyond conventional clinical variables. External validation and assessment of added predictive value will be essential before these microgenderome-related candidate signatures can be considered clinically useful.

#### 3.5.2 Sex-specific interventional strategies for gut microecology

Clinical evidence suggests that microbiome-targeted interventions may influence migraine-related outcomes, but current data are insufficient to establish sex-specific efficacy. In a randomized, double-blind, controlled trial involving women with migraine, a 12-week synbiotic intervention comprising 12 probiotic strains and fructooligosaccharides reduced attack frequency, analgesic consumption, and serum hs-CRP levels (Ghavami et al., 2021). Dietary fibers such as inulin have also been reported to improve migraine-related outcomes and comorbid affective symptoms in women, providing additional preliminary support for prebiotic interventions (Vajdi et al., 2024). These findings suggest that modulation of gut microbial activity, intestinal permeability, and inflammatory tone may influence migraine outcomes in women, but they do not prove a female-specific therapeutic mechanism.

Mechanistic studies and genetic evidence provide useful leads for future microbiome-targeted intervention research. In migraine animal models, manipulation of microbial communities has been associated with changes in trigeminovascular activation and migraine-like behaviors (Fan et al., 2026). Mendelian randomization studies have suggested potential causal links between specific gut microbial taxa, including *Coprococcus* and *Anaerotruncus*, and migraine risk, with circulating inflammatory proteins proposed as possible mediators (Ma et al., 2026). For instance, mediation analyses have suggested that inflammatory pathways may partly account for associations between *Actinobacteria a*nd migraine risk, although these estimates should be interpreted as model-dependent and preliminary. These findings support further investigation of microbial taxa and metabolites as candidate intervention targets, but they should not yet be interpreted as evidence that microbiome-targeted therapies modify the underlying disease process.

In summary, microbiome-targeted interventions show preliminary promise as adjunctive strategies for migraine management, but their clinical implementation requires rigorous validation, standardized protocols, and careful consideration of biological sex, hormonal status, baseline microbial function, and inflammatory or barrier-related phenotypes. It is important not to frame prebiotics, synbiotics, probiotics, dietary modification, or fecal microbiota transplantation (FMT) prematurely as sex-specific therapies. A more appropriate goal is to determine whether biological sex, hormonal status, reproductive stage, gut barrier function, inflammatory activation, and baseline microbial features modify treatment mechanisms and clinical responses.

More intensive microbiome-modifying approaches, especially FMT, should be discussed with particular caution. Although FMT has attracted interest in several microbiome-related disorders, its application in migraine remains experimental and is not supported by sufficient migraine-specific clinical evidence. Potential risks include transmission of infectious agents, donor-dependent variability, unpredictable ecological effects, and uncertain long-term safety. Therefore, any future investigation of FMT or defined microbial consortia in migraine should require rigorous donor screening, standardized manufacturing and quality control, regulatory oversight, adverse-event monitoring, and carefully designed clinical trials before clinical application can be considered.

## 4 Limitations

While research into the gut microecology of migraine has made preliminary progress from a sex-based perspective, the clinical translation of existing evidence faces multiple limitations and challenges.

First, methodological constraints limit the generalizability of current conclusions. Most clinical studies feature relatively limited sample sizes and generally lack rigorous sex-stratified analysis. Even in studies encompassing both male and female participants, researchers frequently fail to report results separately for each sex subgroup, making it difficult to evaluate whether interventions have sex-related differences in efficacy.

Second, the systematic control of confounding factors remains inadequate. Variables such as dietary habits (Schoeler et al., 2024; Zeng et al., 2024), medication exposure – particularly the frequent use of non-steroidal anti-inflammatory drugs (NSAIDs) (Barekat et al., 2026; Zádori et al., 2023) – hormonal status including menopause (Vriend et al., 2024), and gastrointestinal comorbidities (Meng et al., 2025; Svolos et al., 2021) may substantially influence microbial composition.

Additionally, limitations exist in study design and biological translation. Current clinical research relies heavily on cross-sectional designs or short-term interventions, lacking longitudinal dynamic monitoring aligned with the migraine disease course and hormonal cycles. Consequently, establishing a clear causal link between dysbiosis and headache attacks remains challenging. Although animal and non-migraine disease models suggest that androgen-related microbiota signaling and sex-related tryptophan metabolic responses may influence neuroimmune regulation, extrapolation of these mechanisms to human migraine requires caution because of interspecies heterogeneity, disease-context specificity, and differences in hormonal regulation and microbial colonization.

Finally, the gut–brain axis in migraine represents a complex network involving interactions among endocrine, immune, microbial, metabolic, and neuroactive signaling systems. Current research often focuses on isolated dimensions and has not fully integrated the interactions among sex hormones, microbiota, and metabolites within the microgenderome framework (Hokanson et al., 2024; Wu et al., 2024). Therefore, future research should employ prospective cohorts, sex-balanced designs, and integrated multi-omic analyses. By strictly controlling for confounding variables, these approaches may improve the reliability of findings and clarify their potential relevance for sex-stratified biomarkers and adjunctive therapeutic strategies.

## 5 Conclusion and future directions

**FIGURE 6:**
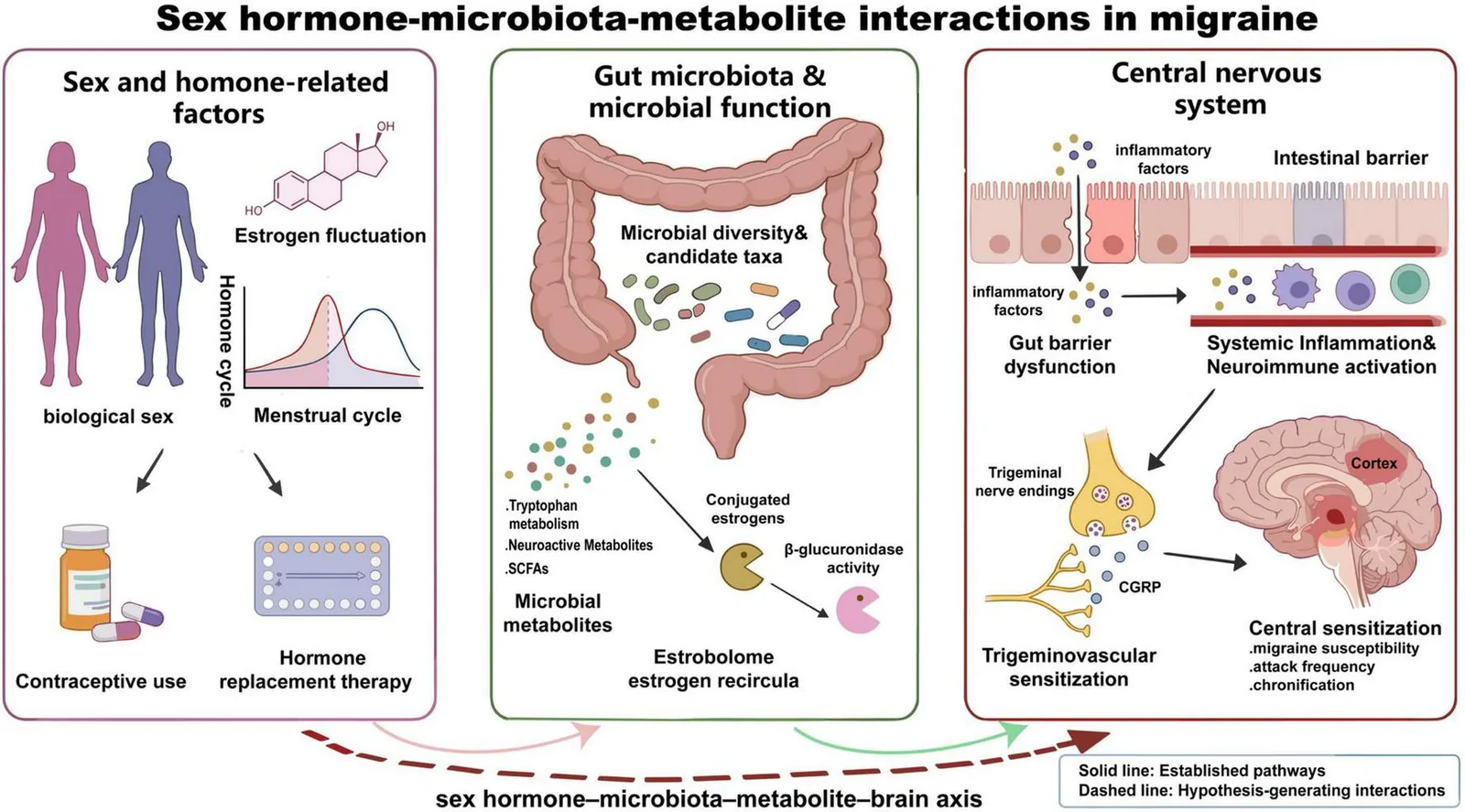
Hypothesis-generating microgenderome framework in migraine. This Figure illustrates a hypothesis-generating framework in which biological sex and hormone-related variables may shape gut microbial composition and function, while microbial enzymes and metabolites may influence estrogen recirculation, gut barrier integrity, inflammatory tone, neuroimmune signaling, and trigeminovascular activation. Solid arrows indicate pathways supported by migraine-specific or adjacent mechanistic evidence; dashed arrows indicate proposed sex-modified mechanisms that require direct validation in migraine. Illustration was created with Biorender.com.

This review synthesizes current evidence suggesting that the gut microbiota and metabolic profiles of migraine patients may differ according to biological sex. These differences support a hypothesis-generating sex hormone–microbiota–metabolite–brain framework that may help explain sex-related differences in migraine susceptibility, severity, and treatment response; however, this framework should not be interpreted as an established causal mechanism (Figure 6).

Regarding microbial composition, female migraine cohorts have shown taxonomic shifts characterized by a reduction in the abundance of taxa such as *Clostridia* and *Ruminococcus*, alongside an enrichment of genera with pro-inflammatory potential, including *Desulfovibrio* and *Gemmiger* (Vuralli et al., 2024a). At the metabolic level, alterations in the tryptophan–kynurenine pathway, particularly KYNA/QUIN-related changes, and possible differences in short-chain fatty acid profiles may provide candidate neurochemical pathways for future sex-stratified validation (Lanza et al., 2022; Liu J. et al., 2024; Sriprasert et al., 2025). Furthermore, intervention trials involving female cohorts provide preliminary evidence that modulation of the gut microecology may alleviate migraine symptoms and influence inflammatory tone or intestinal permeability, but they do not yet establish sex-specific therapeutic efficacy (Ghavami et al., 2021; Tereshko et al., 2023; Vajdi et al., 2024).

The potential mechanisms underlying these phenomena may involve bidirectional feedback regulation between sex hormones and the microbiota, heterogeneous responses within immune–neuroinflammatory pathways, and indirect modulation of pain processing by microbiota-related neuroactive metabolites. Although a preliminary mechanistic framework has emerged, the field remains constrained by a predominance of cross-sectional designs, which limit causal inferences, and insufficient control over confounding variables such as diet, medication, and fluctuating hormonal states.

To address these limitations, future research should prioritize several critical dimensions. First, large-scale, sex-balanced, prospective longitudinal cohort studies are needed to test temporal associations between microbial dysbiosis and migraine progression. Second, investigations should explore the dynamic evolution of the microgenderome across different physiological phases, including the menstrual cycle and menopause. Finally, the integration of multi-omic data should be leveraged to evaluate exploratory sex-stratified candidate markers, including KYNA/QUIN-related measures, zonulin, oral microbiome classifiers, SCFAs, inflammatory mediators, and hormone-related microbial functional genes. Such studies may provide an evidence-based foundation for constructing biologically informed, sex-stratified diagnostic and adjunctive therapeutic strategies for migraine management.
